# A cross-sectional investigation of *Leptospira* at the wildlife-livestock interface in New Zealand

**DOI:** 10.1371/journal.pntd.0011624

**Published:** 2023-09-06

**Authors:** Marie Moinet, Hedwich Oosterhof, Shahista Nisa, Neville Haack, David A. Wilkinson, Danielle Aberdein, James C. Russell, Emilie Vallée, Julie Collins-Emerson, Cord Heuer, Jackie Benschop

**Affiliations:** 1 Tāwharau Ora School of Veterinary Science, Massey University, Palmerston North, New Zealand; 2 Faculty of Veterinary Medicine, Utrecht University, Utrecht, The Netherlands; 3 New Zealand Food Safety Science & Research Centre, Hopkirk Research Institute, Palmerston North, New Zealand; 4 School of Biological Sciences and Department of Statistics, University of Auckland, New Zealand; University of Kentucky College of Medicine, UNITED STATES

## Abstract

There has been a recent upsurge in human cases of leptospirosis in New Zealand, with wildlife a suspected emerging source, but up-to-date knowledge on this topic is lacking. We conducted a cross-sectional study in two farm environments to estimate *Leptospira* seroprevalence in wildlife and sympatric livestock, PCR/culture prevalence in wildlife, and compare seroprevalence and prevalence between species, sex, and age groups. Traps targeting house mice (*Mus musculus*), black rats (*Rattus rattus*), hedgehogs (*Erinaceus europaeus*) and brushtail possums (*Trichosurus vulpecula*) were set for 10 trap-nights in March-April 2017 on a dairy (A) and a beef and sheep (B) farm. Trapped wild animals and an age-stratified random sample of domestic animals, namely cattle, sheep and working dogs were blood sampled. Sera were tested by microagglutination test for five serogroups and titres compared using a Proportional Similarity Index (PSI). Wildlife kidneys were sampled for culture and qPCR targeting the *lipL32* gene. True prevalence in mice was assessed using occupancy modelling by collating different laboratory results. Infection profiles varied by species, age group and farm. At the MAT cut-point of ≥ 48, up to 78% of wildlife species, and 16–99% of domestic animals were seropositive. Five of nine hedgehogs, 23/105 mice and 1/14 black rats reacted to *L*. *borgpetersenii* sv Ballum. The sera of 4/18 possums and 4/9 hedgehogs reacted to *L*. *borgpetersenii* sv Hardjobovis whilst 1/18 possums and 1/9 hedgehogs reacted to Tarassovi. In ruminants, seroprevalence for Hardjobovis and Pomona ranged 0–90% and 0–71% depending on the species and age group. Titres against Ballum, Tarassovi and Copenhageni were also observed in 4–20%, 0–25% and 0–21% of domestic species, respectively. The PSI indicated rodents and livestock had the most dissimilar serological responses. Three of nine hedgehogs, 31/105 mice and 2/14 rats were carrying leptospires (PCR and/or culture positive). True prevalence estimated by occupancy modelling in mice was 38% [95% Credible Interval 26, 51%] on Farm A and 22% [11, 40%] on Farm B. In the same environment, exposure to serovars found in wildlife species was commonly detected in livestock. Transmission pathways between and within species should be assessed to help in the development of efficient mitigation strategies against *Leptospira*.

## Author summary

Leptospirosis is a bacterial disease that can be transmitted through the urine of infected animals. Recently, the number of human cases of leptospirosis in New Zealand has increased, and wildlife is suspected to be an emerging source. To better understand this, a study was conducted on two farms in New Zealand. Wild animals, including mice, rats, hedgehogs, and possums, and domestic animals, including cattle, sheep, and working dogs, were captured, and tested for exposure to *Leptospira*. We found multiple serovars of leptospirosis throughout both livestock and wildlife, with important variation by species and age class. Reactions to Ballum were found in all domestic and wild species and all ages while domestic animals, possums, and hedgehogs commonly reacted to Hardjobovis. Cattle, sheep, hedgehogs, and possums also showed exposure to Tarassovi. These indicate potential multiple and complex pathways of disease transmission and dynamics among serovars and suggest that *Leptospira* infection in wildlife in New Zealand can pose a risk to both livestock and human health. Further research is needed to understand how the bacteria are being transmitted to effectively prevent the associated disease.

## Introduction

Leptospirosis is a zoonosis caused by pathogenic bacteria of the genus *Leptospira* affecting a wide range of vertebrates worldwide. The advent of genomic methods has shed light on this complex genus [1], with 71 different species and more than 300 serovars currently described [2–5], a number likely to increase. In New Zealand (NZ), only two species and six serovars (sv) are known to be endemic in animals: *L*. *borgpetersenii* sv Hardjobovis, Ballum, Balcanica and Tarassovi and *L*. *interrogans* sv Pomona and Copenhageni. *L*. *interrogans* sv Canicola and Australis have also been isolated but from human cases only [6].

Often overshadowed by the dominance of rodents in the literature, the recognition of livestock as an important source of leptospirosis is increasing worldwide [7–10]. In NZ the situation is the opposite: this zoonosis has a clear occupational pattern, with more than two-thirds of notified cases being farm or abattoir workers [11]. Cattle, pigs, and subsequently sheep and farmed deer have been shown to maintain serovars Hardjobovis or Pomona independently of wildlife [12–15]. These two serovars were responsible for 99% of notified human cases in the 1970s [16]. The uptake of vaccination in the pig and dairy industries in the 1980s combined with other hygienic measures was followed by a rapid and sustained reduction of human cases from these serovars [17].

Several studies in the 1950s – 1970s investigated NZ wildlife as a potential reservoir of *Leptospira* [18]. Black rats (*Rattus rattus*), brown rats (*R*. *norvegicus*), house mice (*Mus musculus*) and hedgehogs (*Erinaceus europaeus*) were described as maintenance hosts for Ballum and possums (*Trichosurus vulpecula*) for Balcanica [19]. In contrast to numerous countries worldwide, rodents and wildlife at the time were identified as a minor public health concern for leptospirosis in NZ [20–22]. At the time, Ballum was rarely reported (< 1%) in the notified human cases and thus did not warrant investment into research on *Leptospira* infection in wildlife [18].

Concurrent to the studies on wildlife mentioned above, studies conducted on livestock underlined the scarcity of reactions to serovars other than Hardjobovis and Pomona [12]. As reviewed in [18], titres against Tarassovi, Ballum or Copenhageni were scarce (for instance 3.5% seroprevalence (17/480) to Ballum in cattle at the 1:17 titre cut-off [12]) and at that time interpreted as cross-reactions [19]. Hathaway et al. [23] described this epidemiological situation through the concept of nidality, where different serovars were considered to evolve in adjacent ‘niches’ with virtually no spillover between domestic and wild species. Subsequent studies focused on Hardjo and Pomona, and other serovars were omitted from test panels.

Although spillovers were formerly considered to be rare events in NZ, this may not be the case. Vaccinated dairy cattle have recently been shown to shed serovars not traditionally associated with cattle [24]. Other strains are now more common among human cases, especially Ballum, responsible for 30% of the cases notified in 2015 and 2016 [11, 25]. Livestock-associated occupations still predominate human case notifications although Ballum is found in farmers but not meatworkers and is strongly associated with other (non-livestock-associated) occupations [26]. It is unknown if this change in livestock and human epidemiology is due to an increased exposure to maintenance hosts shedding those emerging serovars or a change in the role of livestock in maintaining them. Current information on all serovars circulating in wildlife and livestock is needed.

The objectives of this cross-sectional study were to (1) estimate the seroprevalence in wildlife and sympatric livestock in two farm environments; (2) estimate PCR/culture prevalence in wildlife on those farms; (3) compare seroprevalence and prevalence between species, sex, and age groups on the two farms, and (4) estimate the true prevalence of natural *Leptospira* infection in mice using occupancy models.

## Materials & methods

### Ethics statement

The present research was done in accordance with the NZ Animal Welfare Act 1999 and the Massey University Code of Ethical Conduct. The Massey University Animal Ethics Committee approved the procedures done on animals under the protocol 16/93.

### Study sites

We selected two farms where *Leptospira* infection had been detected in livestock during previous studies. Farm A was a dairy farm in coastal Manawatū, identified after an outbreak of leptospirosis due to sv Hardjobovis and Pomona among the farm workers [27]. Livestock from this farm had been investigated and an intervention study had been conducted to assess the effectiveness of livestock vaccination to decrease the shedding rates of sv Hardjobovis and Pomona [28]. This study underlined the risk posed by two non-vaccine serovars, Ballum and Tarassovi [28]. Farm A comprised two dairy herds of 228 and 400 milking cows, 150 rising 1-year-olds (R1) and 150 rising 2-year-olds (R2). The farm spanned 130 ha of lowland pastures and was bounded by a pine forest on one side (average elevation: 20m).

Farm B was a beef & sheep farm in the Tararua region, where sheep naturally exposed to Hardjobovis and Pomona had been studied previously [29]. It comprised *ca*. 2600 ‘hoggets’ (1-year), 2300 ‘2-tooth’ (2-year) and 4000 mixed-age ewes, 350 calves, 160 R1 and 100 R2 heifers and 300 mixed-age cattle. Around 20 working dogs were also present on Farm B that spanned 2100 ha of hilly pastures bordered with native bush (average elevation: 444 m).

Before the arrival of Māori and European settlers, NZ was an archipelago devoid of terrestrial mammals except for two species of bats [30]. Humans subsequently purposefully and inadvertently introduced many mammal species to New Zealand. Pastoral farming systems now prevail and a limited number of imported cultivars dominate, like perennial ryegrass (*Lolium perenne*) and white clover (*Trifolium repens*) [31]. All mammals present in the studied farms are thus an eclectic assortment of introduced species that co-exist in highly modified ecological systems.

### Fieldwork

#### Wildlife trapping

In each site, 72 Longworth small mammal live-traps (Penlon Ltd., Oxford, UK) targeting mice, 45 Tomahawk 202.5 collapsible live-traps (Tomahawk Live Trap, Hazelhurst, WI, USA) targeting rats, and 36 Havahart #1099 live-traps (Woodstream Corp., Lititz, PA, USA) targeting possums and hedgehogs were used. The three types of traps were set on grids in locations favourable to rodents, possums, and hedgehogs with approximately 10 m, 25 m, and 50 m spacing, respectively [32–34] and their GPS positions were recorded. These traps were set in March-April 2017 for up to 10 days per site, baited with peanut butter, cat food and pieces of apple covered with a mixture of sugar and cinnamon and checked daily.

#### Wildlife sampling

Trapped wild animals were anesthetised, a blood sample taken, and animals were then euthanised for organ sampling as described by Herbreteau et al. [35]. Mice and rats were anaesthetised using isoflurane (Attane, Bayer) insufflated in a plastic bag and euthanised while sedated by cervical dislocation. Other species were anaesthetised by intramuscular injection of a mix of medetomidine (Domitor, Zoetis NZ Ltd, 50 to 150 μg/kg) and ketamine (Phoenix pharm, 5 to 10 mg/kg) and euthanised while sedated with pentobarbital (Pentobarb 300, Provet, 150 mg/kg). In addition to blood, a kidney was sampled aseptically for culture and PCR. Concomitantly, other organs (remaining kidney, spleen, liver, lung, heart, brain, gastrointestinal tract) were collected and stored in cryotubes and formalin for other studies. Species, sex, reproductive status, and weight were recorded for each trapped animal. Age (juvenile, sub-adult, or adult) was determined according to the weight and reproductive status. In addition, two Sambar deer hunted during the trapping session on Farm B boundaries and one by-catch feral cat from each farm were also sampled and samples processed similarly.

#### Livestock sampling

Healthy domestic animals were sampled by mob according to the farmers’ schedule (when animals were gathered for milking, drenching, shearing, pregnancy testing or annual vaccine booster, Table 1). We expected a seroprevalence of 20% for dogs (which was the seroprevalence observed in working dog breeds [36]), 80% in sheep and beef cattle (which was the seroprevalence observed in beef & sheep farms when *Leptospira* was present [37], and 50% in dairy cattle (which was the seroprevalence in the dairy farm at first sampling [27]). Assuming the proportions of seropositive animals in each farm and group were as expected, and adjusting for a finite population, we used the formula for estimating the expected seroprevalence with 10% absolute precision and 95% confidence in [38] to calculate the sample size in each species or age-group (Table 1). Blood was collected by caudal or jugular venipuncture using a one-inch 20 G vacutainer needle and a CAT Plus Blood Collection Tube without anticoagulant (BD Vacutainer). Blood samples were transported on ice in a cooling box to the Molecular Epidemiology and Public Health Laboratory (^*m*^EpiLab, Massey University, Palmerston North), where they were centrifuged at 2000 *g* for ten minutes to obtain serum. A convenience sample of urine was collected for culture from cattle and sheep voiding urine during blood sampling. Urination was otherwise stimulated by tickling the vulva and mid-stream urine samples were collected in 60 mL sterile containers. A sample size of 30 urine samples per species and age-group was targeted. Information on vaccination status was also retrieved from the farmers.

**Table 1:** Number of domestic animals sampled per age group and farm.

	Group	Expected seroprevalence (%)	Approximate group size (#)	Sample size (#)	Sampling date
Farm A	Milking cows	50	250	70	29/03/2017
	1-year-old dairy cattle (R1)		150	59	10/04/2017
	2-year-old dairy cattle (R2)		150	59	10/04/2017
Farm B	Working dogs	20	25	18	23/05/2017
	1-year-old ewes (hoggets)	80	2600	61	23/05/2017
	2-year-old ewes (2-tooths)		2300	60	5/04/2017
	Mixed-age ewes		4000	61	10/03/2017
	1-year-old beef cattle (R1)		160	45	\
	2-year-old beef cattle (R2)		100	39	5/04/2017
	Mixed-age beef cattle		300	52	\

\: not sampled

### Culture

To keep contamination to a minimum, cultures were processed on-farm and the method adapted accordingly. A field lab was set up to process all wild animal samples, while livestock samples were processed next to the sampling area (yard, milking shed or paddock). Kidneys to be cultured were removed aseptically within half an hour of euthanasia. Kidneys were washed with 70% ethanol and flamed, 1 cm^3^ (or the whole kidney if less) was placed with 1 mL (or an equivalent volume) of Phosphate Buffered Saline (PBS) in a sterile Petri dish and dilacerated using a sterile scalpel blade. An approximate 0.5 to 1 mL aliquot of the kidney slurry was saved into a cryotube for molecular analysis. The remaining kidney and PBS slurry was pipetted into a tube with 2 mL PBS and left to stand for approximatively 30 minutes. A culture vial containing 5 mL EMJH + 5’-fluorouracil was then inoculated with 0.5 mL of liquid and two subsequent serial dilutions (1/10) were made to limit potential culture contamination. Similarly, livestock urine was first collected in a sterile container, and, within 1 h after collection, 0.5 mL was inoculated in 5 mL EMJH + 5’-fluorouracil with two subsequent serial dilutions (1/10). Culture vials of all dilutions were stored at ambient temperature and protected from the light in the field and placed at 28°C on a shaker in an incubator as soon as they reached the ^*m*^EpiLab. They were checked under the dark field microscope at least every two weeks for 14 weeks. Cultures that had to be discarded before 14 weeks due to contamination were deemed inconclusive.

### Laboratory analyses

All samples were processed by the same laboratory personnel.

#### MAT

Microscopic agglutination test (MAT) was used to test all sera for antibodies against *Leptospira borgpetersenii* sv Hardjobovis, Ballum and Tarassovi and *Leptospira interrogans* sv Pomona and Copenhageni (Table 2), covering all serogroups endemic to New Zealand. The technique used was described by Fang et al. [39]. Two-fold dilutions of the serum sample ranging from 1:24 to 1:3072 were made in 0.9% sterile saline for each serovar. After being incubated for 1.5–4 h with a volume of live antigen suspension of each of the above-mentioned serovars, the presence of agglutination was checked under a dark-field microscope. A positive control (standard antiserum, World Health Organisation (WHO) Leptospirosis Reference Centre, Amsterdam) and negative control (0.9% saline) were included for each serovar tested, on each day of testing. The endpoint of an agglutination reaction was deemed to be the dilution at which approximately 50% of *Leptospira* had agglutinated and expressed as a reciprocal titre (*e*.*g*. titre 24 for dilution 1:24). Since this serological test was used to assess previous exposure (seroprevalence) to leptospires at the population level, and not for clinical diagnosis, the positive threshold was set at a titre of 48 or higher [40].

**Table 2:** Strains of *Leptospira* included in the Microscopic Agglutination Test.

Genomospecies	Serogroup	Serovar	Strain
*L*. *interrogans*	Icterohaemorrhagiae	Copenhageni	M20
	Pomona	Pomona	68*
*L*. *borgpetersenii*	Sejroë	Hardjobovis	180*
	Ballum	Ballum	Mus 127
	Tarassovi	Tarassovi	Perepelitsin

* ^**m**^ **EpiLab strains**

#### *lipL32* real-time PCR on kidney

DNA from 80 μL of the kidney PBS slurry was extracted with QIAamp DNA mini kit (Qiagen, Bio-Strategy Ltd, Auckland, NZ). A real-time Polymerase Chain Reaction (PCR) assay was performed on each extract using a probe targeting the *LipL32* gene, only present in the pathogenic clade of the genus *Leptospira* [41]. Reactions were performed in a total volume of 10 μL consisting of 0.4 μM each of forward and reverse primers, of sequences 5’-AAG CAT TAC CGC TTG TGG TG-3’ (*lipL32*-45-F) and 5’-GAA CTC CCA TTT CAG CGA TT-3’ (*lipL32*-286-R), 0.13 μM of probe, of sequence FAM-5’-AA AGC CAG GAC AAG CGC CG-3’-BHQ1 (*lipL32*-189P), 2 μL of ToughMix (Quantabio), 2 μL PCR grade water and 2 μL of DNA template and analysed as described previously [41, 42]. We used a Qiagen Rotor-Gene Q machine (Bio-Strategy Ltd, Auckland, NZ), PCR grade water as a negative control and DNA extracted from approximately 3 × 10^8^ cells/mL of pure culture of *L*. *borgpetersenii* serovar Hardjobovis as a positive control. Reactions with a Cq ≤ 37 were considered positive.

### Data analysis

Unless otherwise stated, all analyses were conducted in ℝ version 3.4.2 [43]. We differentiated the seroprevalence (estimated by MAT), the prevalence (estimated by culture or PCR) and the true prevalence (the proportion of animals exposed to *Leptospira* infection). The probability of shedding amongst seropositive and seronegative wild animals was calculated by dividing the number of animals positive for PCR and/or culture by the total number of animals tested within each stratum.

#### Confidence Intervals for proportions

Exact confidence intervals (CI) of observed culture and PCR prevalence and seroprevalence were computed based on the binomial distribution [38].

#### Geometric Mean Titres (GMT)

The geometric mean titre of positive sera (GMT) and all sera (GMT0) was calculated using the formulae given in [37]. While sera for which no antibodies were detected (titre <24) were excluded from the calculation of the GMT, they were given a log-titre of 0 and included in the calculation of the GMT0.

#### True prevalence

Misclassification bias can arise from the use of imperfect tests giving false-positive and false-negative results. To limit this bias, true prevalence in mice was computed for each farm using site-occupancy modelling as in [44]. Occupancy models are widely used in ecology to estimate the proportion of sites occupied by an animal species while accounting for imperfect detection. Considering animals as sites occupied or not by a pathogen, these models can be adapted to infer the probability *ψ* an animal is ‘occupied’—*i*.*e*. the true prevalence of infection—and the probability *p* of pathogen detection conditional on the pathogen presence—*i*.*e*. the sensitivity of the test(s) used [44]. Results of the three laboratory diagnostic methods used in this study (MAT, PCR and culture) were considered as detection occasions and for each mouse, a ‘detection history’ (*i*.*e*., an observed status being a combination of the three test results for a given mouse) was built to fit Hidden Markov Models to estimate the true prevalence *ψ* of *Leptospira* infection in mice. For instance, an animal with a positive MAT and PCR and negative culture had a ‘detection history coded as ‘110’ (out of a total of eight possible observed statuses). Hidden Markov Models were implemented in software E-Surge version 2.1.2 as described by [45]. While *ψ* was allowed to vary between farms, *p* was considered either constant across laboratory methods or method-specific. Model selection was based on QAICc (Quasi Akaike Information Criterion corrected for small sample size and adjusted for overdispersion) [44]. The model with the lowest QAICc was selected as the model that fitted the data best. An important assumption of this method was that all animals tested positive for any given test were considered as true positives (perfect specificity). Mice with missing data for at least one laboratory method were not included. Details on occupancy models and their parameterisation are presented in S1 Appendix. True prevalence was also estimated using Bayesian latent class modelling, a second method not assuming perfect specificity, and the results compared (S2 Appendix).

#### Kappa test for cross-reaction

Agreement beyond chance between MAT results for different serovars was tested using Kappa (κ) tests for all wild and all domestic species. A Cohen’s Kappa and a square-weighted Kappa were calculated respectively for each pair of MAT serovar results (positive/negative) and each pair of MAT serovar log-titres. While Cohen’s Kappa is adapted to binary data, the square-weighted Kappa gives more weight to bigger differences between titres (*e*.*g*. titres 24–3072 *vs*. 24–48) and is therefore more adapted for ordinal data. Values κ ≤ 0 indicated no agreement while the strength of agreement was considered as poor for 0.01 ≤ κ < 0.2, fair for 0.2 ≤ κ < 0.4, moderate for 0.4 ≤ κ < 0.6, good for 0.6 ≤ κ < 0.8 and very good for κ ≥ 0.8. Good or very good agreement would suggest cross-reaction.

#### PSI-Czekanowski index

A proportional similarity index (PSI) in the serological responses between species was computed for all species. The PSI or Czekanowski index has first been used to measure the breadth of a population’s niche in ecology, but also to measure the similarity between the frequency distributions of pathogen types among different animal species [46, 47]. It is calculated as $PSI=1-0.5\sum_{i}|p_{i}-q_{i}|=\sum_{i}min(p_{i},q_{i})$ where *p*_*i*_ and *q*_*i*_ are the proportion of serovar *i* out of all serovars detected in animal species P and Q respectively. The same positivity threshold as MAT was used (titre ≥ 48). The closer the PSI is to 1, the more similar are the frequency distributions of *Leptospira* serovars between two species; the closer the PSI is to 0, the more dissimilar they are. We determined 95% credible intervals using a bootstrap simulation method with 2000 replications [48]. If the credible intervals included 0.5 the PSI was regarded as inconclusive. A high PSI would suggest that serovars are transmitted between two host species, while a PSI of zero indicates no overlap in circulating serovars.

## Results

### Species composition in the different study sites

There were respectively 720, 430, and 351 trap-nights for Longworth, Tomahawk, and Havahart traps on Farm A and 648, 418, and 332 trap-nights on Farm B. Rats, hedgehogs, and possums were trapped in both Tomahawk and Havahart traps. The number of animals captured per 100 trap-nights and sampled in each farm are detailed in Table 3. No possums were trapped on Farm A where possum control had been in place within the farm and the neighbouring forest for several years. Other wild mammals not targeted by the traps were also observed while on site: feral cats (*Felis catus*) and rabbits (*Oryctolagus cuniculus*) on both farms, Sambar deer (*Cervus unicolor*) on Farm A and red deer (*C*. *elaphus*) on Farm B. All serology, culture, and PCR tests were negative for the two Sambar deer and two by-catch feral cats sampled. We did not have the opportunity to sample the R1 and mixed-age beef cattle on Farm B during the study (Table 4).

**Table 3:** Wildlife abundance (A) in captures per 100 trap-nights, total number of animals sampled (N) and with a positive result (bolded) for *Leptospira* infection by Culture, PCR or Microscopic Agglutination Test (MAT; cut-off 48).

	Species	A	N	Culture # positive (% [95% CI])	PCR # positive (% [95% CI])	MAT # positive (seroprevalence [95% CI]) and GMT0 (GMT)					
						Ballum	Copenhageni	Hardjobovis	Pomona	Tarassovi	Overall
**Farm A**	Hedgehog *Erinaceus europaeus*	1.02*	8	**3** (38% [9, 76])	**3** (38% [9, 76])	**4 (50% [16, 84])** 62 (167)	0 (0% [0, 37]) 17 (24)	**4 (50% [16, 84])** 26 (42)	**2 (25% [3, 65])** 40 (305)	**1 (13% [0, 53])** 19 (29)	**6** (75% [35, 97])
	House mouse *Mus musculus*	14.3	79†	**16** (26% [16, 39])	**23** (31% [21, 43])	**18 (25% [15, 36])** 30 (523)	**1 (1% [0, 7])** 12 (96)	0 (0% [0, 5]) 12 (24)	0 (0% [0, 5]) 0	0 (0% [0, 5]) 12 (24)	**18** (25% [15, 36])
	Black rat *Rattus rattus*	1.02*	3	**1/3**	**1/3**	**1/3** 38 (384)	0/3 0	0/3 0	0/3 0	0/3 15 (24)	**1/3**
**Farm B**	Hedgehog *Erinaceus europaeus*	0.67*	1	0/1	0/1	**1/1**	0/1	0/1	0/1	0/1	**1/1**
	House mouse *Mus musculus*	5.71	33^¶^	**6** (18% [7, 35])	**6** (18% [7, 35])	**5 (16% [5, 33])** 19 (253)	0 (0% [0, 11]) 12 (24)	0 (0% [0, 11]) 13 (24)	0 (0% [0, 11]) 0	0 (0% [0, 11]) 0	**5** (16% [5, 33])
	Black rat *Rattus rattus*	2.40*	11	0 (0% [0, 28])	**1** (9% [0, 41])	0 (0% [0, 28]) 0	0 (0% [0, 28]) 0	0 (0% [0, 28]) 0	0 (0% [0, 28]) 0	0 (0% [0, 28]) 13 (24)	0 (0% [0, 28])
	Brushtail possum *Trichosurus vulpecula*	2.67*	19‡	0 (0% [0, 31])	0 (0% [0, 26])	0 (0% [0, 19]) 12 (24)	0 (0% [0, 19]) 12 (24)	**4 (22% [6, 48])** 34 (272)	0 (0% [0, 19]) 0	**1 (6% [0, 27])** 13 (34)	**5** (28% [10, 53])

The prevalence and 95% confidence interval are indicated in brackets when N>5, and when applicable, the serovar specific geometric mean titre of all sera (GMT0) or of sera with titres ≥ 24 (GMT) are indicated for each serovar.

* Calculated with Tomahawk and Havahart traps since animals were caught in both types

† 1 had PCR only and 17 had PCR and MAT only

¶ 1 had PCR and culture only

‡ 1 had PCR and culture only, 2 had PCR and MAT only, and 7 had MAT only

**Table 4:** Herd size, number of blood (S) and urine (U) sampled from domestic species and with a positive result (bolded) for *Leptospira* infection by Microscopic Agglutination Test (MAT; cut-off 48).

	Group	Herd size	S	U	MAT* # positive (seroprevalence [95% CI]) and GMT0 (GMT)					
					Ballum	Copenhageni	Hardjobovis	Pomona	Tarassovi	Overall
**Farm A**	R1 Dairy cattle	150	60	30	**7 (12% [5, 23])** 19 (39)	**3 (5% [1, 14])** 14 (114)	**18 (30% [19, 43])** 23 (35)	**10 (17% [8, 29])** 17 (44)	0 (0% [0, 6]) 14 (24)	**26 (43% [31, 57])**
	R2 Dairy cattle	150	60	30	**12 (20% [11, 32])** 21 (34)	0 (0% [0, 6]) 13 (24)	0 (0% [0, 6]) 13 (24)	**2 (3% [0, 12])** 13 (68)	**7 (12% [5, 23])** 21 (28)	**19 (32% [20, 45])**
	Milking cows	228	83	30	**7 (8% [3, 17])** 16 (30)	**1 (1% [0, 7])** 13 (26)	**66 (80% [69, 88])** 66 (72)	**58 (70% [59, 79])** 98 (136)	**8 (10% [4, 18])** 18 (40)	**82 (99% [93, 100])**
	TOTAL Dairy cattle	528	203	90	**26 (13% [9, 18])**	**4 (2% [1, 5])**	**84 (41% [35, 48])**	**70 (34% [28, 41])**	**15 (7% [4, 12])**	**127 (63% [56, 69])**
**Farm B**	Working dogs	20	14	\	**2 (14% [2, 43])** 18 (36)	**3 (21% [5, 51])** 19 (34)	**9 (64% [35, 87])** 87 (101)	0 (0% [0, 23]) 18 (24)	0 (0% [0, 23]) 14 (24)	**9 (64% [35, 87])**
	R1 Beef cattle	160	\	\	\	\	\	\	\	\
	R2 Beef cattle	100	45	5	**2 (4% [1, 15])** 14 (34)	0 (0% [0, 8]) 0	0 (0% [0, 8]) 14 (24)	**1 (2% [0, 12])** 13 (28)	**4 (9% [2, 21])** 17 (28)	**7 (16% [6, 29])**
	Mixed-age Beef cattle	300	\	\	\	\	\	\	\	\
	Hoggets (1Y)	2600	58	\	**4 (7% [2, 17])** 15 (62)	**8 (14% [6, 25])** 17 (35)	**3 (5% [1, 14])** 15 (384)	**41 (71% [57, 82])** 185 (576)	0 (0% [0, 6]) 13 (24)	**42 (72% [59, 83])**
	2-tooth (2Y)	2300	61	\	**4 (7% [2, 16])** 16 (37)	**5 (8% [3, 18])** 15 (36)	**55 (90% [80, 96])** 267 (352)	**12 (20% [11, 32])** 28 (216)	**9 (15% [7, 26])** 20 (31)	**56 (92% [82, 97])**
	Mixed-age ewes	4000	61	11	**3 (5% [1, 14])** 15 (30)	0 (0% [0, 6]) 13 (24)	**40 (66% [52, 77])** 45 (59)	**25 (41% [29, 54])** 32 (64)	**15 (25% [14, 37])** 22 (34)	**49 (80% [68, 89])**
	TOTAL Sheep	8900	180		**11 (6% [3, 11])**	**13 (7% [4, 12])**	**98 (54% [47, 62])**	**78 (43% [36, 51])**	**24 (13% [9, 19])**	**147 (82% [75, 87])**

*The prevalence and 95% confidence interval are indicated in brackets, and when applicable the serovar specific geometric mean titre of all sera (GMT0) or of sera with titres ≥ 24 (GMT) are indicated for each serovar. \ = not sampled

### Seroprevalence and titres

**Fig 1:**
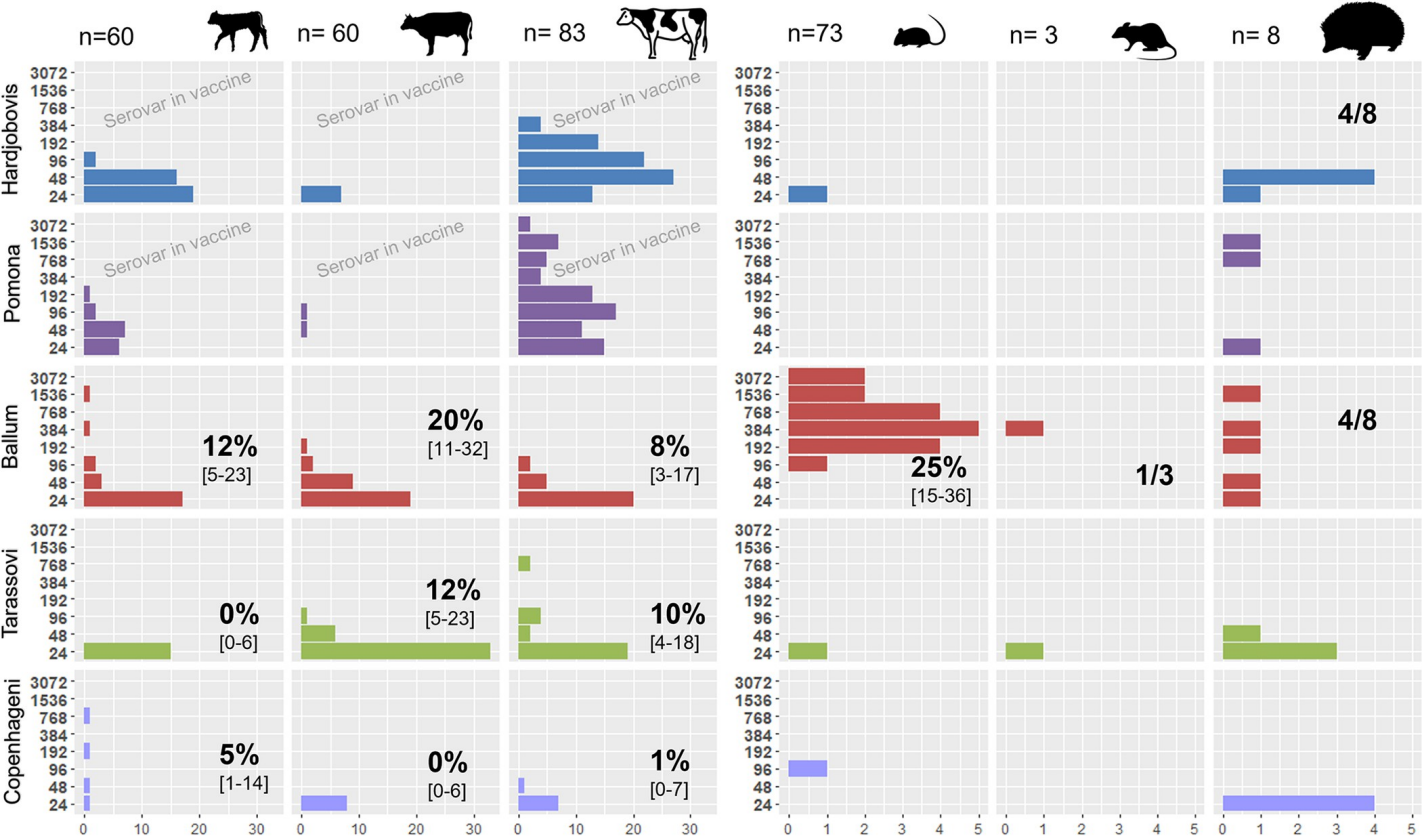
Microscopic Agglutination Test titres observed in domestic and wild animals captured and sampled on Farm A. Seroprevalence (cut-off 48) is also indicated with 95% Confidence Interval. Note the x-axis representing the number of positive animals is on different scales in domestic and wild species. This figure contains open source icons and "Calf silhouette" by Bob Comix under CC BY 4.0 license; "milk cow" by Yu luck and "Mouse" by designer458 under CC BY 3.0 license, "Rat looking right" and "Porcupine shape" from flaticon.com free for personal and commercial use with attribution.

**Fig 2:**
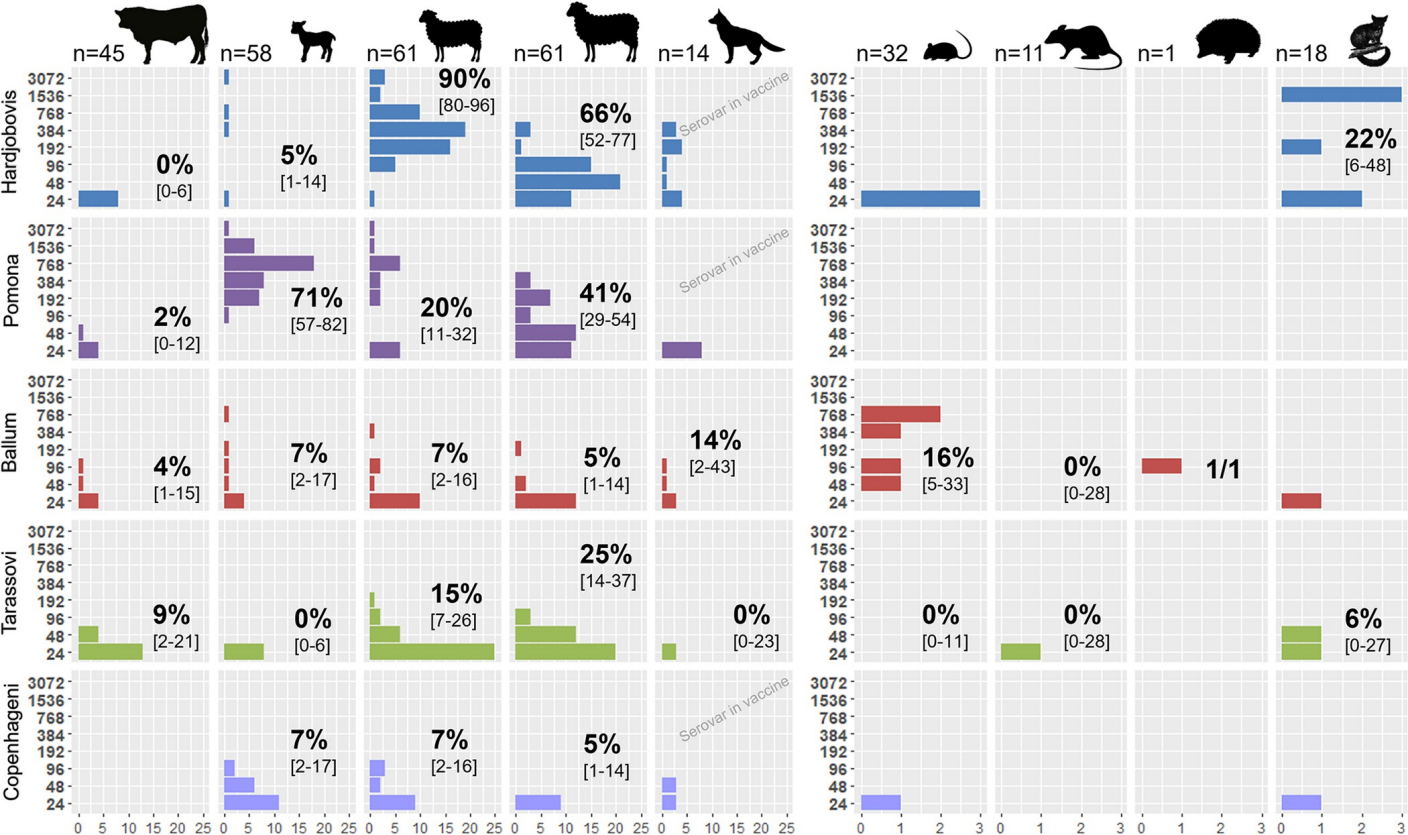
Microscopic Agglutination Test titres observed in domestic and wild animals captured and sampled on Farm B. Seroprevalence (cut-off 48) is also indicated with 95% Confidence Interval. Note the x-axis representing the number of positive animals is on different scales in domestic and wild species. This figure contains open source icons and "Mouse" by designer458, "Lamb" by Shaun Lee and “Sheep” by Hanna Dziewulska under a CC BY 3.0 license; "Rat looking right" and "Porcupine shape" from flaticon.com free for personal and commercial use with attribution.

Serological results are detailed in Table 3 along with culture and PCR results for wildlife and in Table 4 for livestock. According to the farmers, all dairy cattle from Farm A and a majority of dogs from Farm B had been previously vaccinated against leptospirosis—albeit none recently—with a bivalent vaccine (Hardjobovis & Pomona) for the former and an unknown valency for the latter. The distribution of titres in different age groups and species is represented in Figs 1 and 2. Among the unvaccinated livestock, 43% [95% Confidence Interval: 36–51%] and 54% [47–62%] of sheep had titres ≥ 48 for Pomona and Hardjobovis respectively. Only 1/45 R2 beef cattle had a positive reaction for Pomona and none for Hardjobovis. In that group, four had titres of 24 for Pomona and eight for Hardjobovis (Fig 2). No black rats or mice had positive reactions to those two serovars. Four mice had titres of 24 for Hardjo, three from farm B, one from Farm A. In contrast, half of the hedgehogs (4/8) from Farm A had low titres (48) for Hardjo, two had high titres against Pomona (768 and 1536), and four possums from Farm B had titres against Hardjo (192 in a juvenile, and 1536 for three adults).

All groups and species had titres against Ballum except rats and possums on Farm B. Numerous livestock (89/441) had a titre of 24 (Figs 1 and 2). For instance, the seroprevalence for dairy cattle on Farm A increased from 12%, 20% and 8% with a cut-off at 48, to 40%, 52% and 33% with a cut-off at 24 in R1, R2 and milking cows, respectively.

Only livestock were positive for Tarassovi and Copenhageni, with the exception of one possum and one hedgehog for Tarassovi, and one mouse for Copenhageni. As with Ballum, numerous animals (136/441) had a titre of 24 for Tarassovi (Figs 1 and 2). For instance, the seroprevalence for sheep on Farm B increased from 0%, 15% and 25% with a cut-off at 48 to 14%, 56% and 57% with a cut-off at 24 in hoggets, two-tooth and adults, respectively. Except for the pair Copenhageni-Tarassovi that both had a very low seroprevalence, all Kappa calculated for wild species were < 0.2 (Table 5). There was a fair agreement between Ballum and Copenhageni results in domestic species.

**Table 5:** Unweighted Cohen’s Kappa for agreement between Microscopic Agglutionation Test (MAT) results (lower triangle) and weighted (squared weights) kappa for agreement between log-titres (upper-triangle) obtained with the MAT for the 5 different serovars tested.

ALL WILD SPECIES					
	B	C	H	P	T
B	\	0.085	0.114	0.068	0.060
C	0.139	\	0.083	0.168	**0.466**
H	0.135	0.050	\	0.057	0.173
P	0.068	0.097	0.100	\	0.093
T	0.087	**0.380**	0.196	0.136	\
ALL DOMESTIC SPECIES					
	B	C	H	P	T
B	\	**0.339**	0.012	-0.051	0.089
C	**0.215**	\	0.033	0.092	-0.029
H	-0.007	0.014	\	0.043	0.124
P	-0.047	0.055	0.157	\	-0.046
T	0.027	-0.032	0.126	0.019	\

B = Ballum, C = Copenhageni, H = Hardjobovis, P = Pomona, T = Tarassovi; Values ≥ 0.20 are bolded.

The most similar serological responses between host species, as assessed with the PSI calculations, were observed between mice and rats, followed by cattle and sheep (Table 6). Rodents and possums had the most dissimilar serological responses, followed by rodents and sheep.

**Table 6:** Proportional Similarity Index values (lower triangle) and their associated bootstrapped 95% confidence intervals (upper triangle).

	Hedgehog	Mouse	Black rat	Possum	Dog	Sheep	Cattle
Hedgehog	\	0.17–0.67	0.17–0.67	0.08–0.67	0.17–0.74	0.32–0.81	0.39–0.89
Mouse	0.42	\	**0.88–1**	**0–0**	**0–0.4**	**0.03–0.13**	**0.1–0.2**
Black rat	0.42	**0.96**	\	**0–0**	**0–0.36**	**0.02–0.08**	**0.09–0.18**
Possum	0.42	**0**	**0**	\	0.36–0.86	0.38–0.6	0.36–0.56
Dog	0.48	**0.18**	**0.14**	0.64	\	0.44–0.6	0.4–0.61
Sheep	0.63	**0.09**	**0.05**	0.54	0.54	\	**0.81–0.94**
Cattle	0.72	**0.16**	**0.14**	0.5	0.56	**0.91**	\

Values not bolded are values deemed inconclusive (*i.e.* for which the confidence interval includes 0.5)

### Culture and PCR prevalence

Culture and PCR apparent prevalence for wildlife is synthesised in Table 3. All cultures from livestock urine were inconclusive. Additionally, no leptospires were observed under the dark field microscope in those cultures before they were discarded (up to six weeks following sampling).

The occupancy model of best fit as determined by QAICc among those tested (listed in Table 7) was the model with a prevalence *ψ* varying by farm and a probability of detection (or sensitivity) *p* varying according to the laboratory method. This model estimated a true prevalence *ψ* of 38%, [95% credible interval 26–51%] on Farm A and 22% [11–40%] on Farm B. Estimates of test sensitivity were 74% [55–87%] for culture, 88% [69–96%] for PCR and 64% [45–79%] for MAT.

**Table 7:** Model selection for four occupancy models tested in this article. The model of best fit appears bolded

Model	# IPa	Deviance	QAIC	QAICc
**(*ψ*~Farm, *p~*Method)**	**5**	**194.20**	**204.20**	**205.23**
(*ψ*, *p~*Method)	4	196.57	204.57	205.25
(*ψ*~Farm, *p)*	3	199.11	205.11	205.51
(*ψ*, *p)*	2	201.48	205.48	205.68

# IPa = number of identifiable parameters, QAIC(c) = Akaike Information Criterion corrected for small sample size (and adjusted for overdispersion) *ψ* = true prevalence; p = probability of pathogen detection conditional on the pathogen presence

### Probability of shedding

The probability of shedding was 94% (17/18) for seropositive and 9.4% (3/32) for seronegative adult mice, and 60% (3/5) for seropositive and 16% (8/50) for seronegative young mice (juveniles + subadults). This probability was 43% (3/7) for seropositive hedgehogs (all serovars combined). No seronegative hedgehogs were shedding leptospires. Of the nine hedgehogs sampled, seven were adults and two were subadults. The two subadults had low titres (one with 48 for Hardjobovis and the other with 48 for Hardjobovis and Ballum and 24 for Tarassovi) with no leptospires detected or isolated from their kidneys. Two of the 14 rats sampled, both adults, were shedders (14%): one seropositive for Ballum and one seronegative (all sv). The only non-adult rat captured was a subadult negative with all tests. No possums qualified as shedders.

## Discussion

Our study demonstrates that livestock are exposed to serovars that circulate in wildlife in the same environment, especially Ballum. It adds weight to the growing body of evidence coming from surveys on domestic animals and humans that *Leptospira* infection in wildlife in NZ can be a source of infection and a concern for both livestock and public health. Prior to this current study, the most recent studies at the wildlife-livestock interface in NZ were conducted in the late 1970s, four decades ago. Only a small number of cattle and pigs had titres to Ballum, and none above 24 [12, 19, 49]. It was concluded that “despite high prevalence of endemic infection of Hardjobovis and Pomona in cattle and pigs respectively and Ballum and Balcanica in wildlife, [there was] virtually no evidence of interspecies transmission” [23, p. 111]. Today, whether due to changes in vaccination or farming practices, in ecological factors, species distributions or diagnostic techniques, we can see that this is no longer the case. Antibodies against serovars detected in wildlife in our study were commonly found in livestock sharing the same environment, supporting the concept of inter-species disease transmission, or spillover. The small number of serovars known to circulate in NZ allows a better interpretation of *Leptospira* inter-host species epidemiology than in other countries where *Leptospira* diversity is higher.

Spillover has already been described between rodents and livestock. A European study described a higher seroprevalence for serovars associated with house mice and brown rats (Icterohaemorrhagiae, Ballum), in cattle kept indoors where those species were common, and a higher seroprevalence for Grippotyphosa, Australis & Sejroë, which are more associated with other wild rodents, in cattle kept outdoors [50]. In NZ, those other rodents are absent, and mice and rats are present not only in buildings but also in pastures, increasing the infection pressure. In the studied farms, a concomitant assessment of contact frequency using camera trapping confirmed direct and indirect contacts happened between wildlife and livestock [51].

Contrary to livestock, there is a dearth of information about *Leptospira* prevalence or seroprevalence in wildlife in NZ [18]. The sero- and culture prevalences we observed in mice were higher than the two previous estimates assessed in the 1970s. In the first study, seroprevalence was 3% (2/67, titre cut-off of 100) and culture prevalence was 13% (9/67) [52]. In the second study, seroprevalence was 8% (3/39, cut-off 24), and culture prevalence was 16% (11/70) [19,20]. Differences observed could be due to a real difference in prevalence, or due to the methods used. Both previous studies mentioned used a mix of snap-traps and cage-traps to catch mice and rats, and in Brockie [52], blood for the MAT was extracted from hearts preserved in a saline solution and samples taken up to 24h after death. Hathaway [19], mentioned contamination of kidneys from mice caught in snap-traps was a problem. This could reduce the sensitivity of both culture and MAT. Nevertheless, the higher farm prevalence of Ballum in mice by all tests (MAT, PCR, culture) in our study (16–31%) suggests a possible increase over time.

The interpretation of serological results is often challenging. Individuals infected with leptospires will first develop IgM antibodies, and IgG antibodies later, that have a longer half-life. MAT results are further complicated by cross-reactions between serovars and serogroups, especially in acute-phase samples [53]. Some individuals can present paradoxical reactions, where the highest titres are for a serogroup different than the infecting serovar; or anamnestic responses, where the early titres are predominantly against a serovar from a previous exposure [53]. Even if the possibility of cross-reactions can hinder individual diagnosis, MAT results at the population level can give an overview of the serogroups circulating [53].

In the past, cattle titres against Tarassovi, Ballum and Copenhageni have been interpreted as being mainly due to cross-reactions [12, 23]. In our study, R2 (unvaccinated) beef cattle were negative for Hardjobovis or Pomona but positive for Ballum or Tarassovi, making cross-reactivity less likely and true Ballum or Tarassovi infection more likely. The poor strength of agreement between the different MAT serovar results indicated that cross-reactivity was of little consequence and titres more likely to be indicative of previous exposure to several serovars, except possibly for Ballum and Copenhageni in domestic species. This is consistent with an experimental study on calves infected with Pomona, Hardjo, Ballum or Copenhageni, where notable cross-reactions were observed only between Ballum and Copenhageni up to 8 weeks following inoculation [54]. The interpretation of titres in dogs was further hindered by the absence of information on the vaccine type used for working dogs. Although all vaccines licensed for dogs in NZ only cover the serogroup Icterohaemorrhagiae (*i*.*e*. sv Copenhageni), it is possible farm workers administered an off-label cattle trivalent vaccine (Hardjo, Pomona and Copenhageni) on dogs from Farm B as this practice is suspected to be common for working dogs [55, 56]. Titres against Hardjo in possums were likely indicative of an exposure to Balcanica, another serovar in serogroup Sejroë that this species harbours [19], but there were no isolates to confirm the infecting serovar.

Furthermore, the duration of detectable antibodies following infection by a serovar varies according to the host species and serovar, and the rates of re-exposure to this serovar [37]. Vaccinal titres fade more rapidly than titres following natural infection [57]. There is also considerable individual variation in antibody decay [58]. In a cross-sectional study like this, the timing of infection is unknown, and animals previously infected with antibodies titres below the detection limit cannot be distinguished from animals never exposed [59].

On the other hand, culture and PCR methods add information about the true infection status. In this study, 35% of shedding mice (11/31) and one of two shedding rats had no detectable titres (hereinafter referred to as ‘silent shedders’). While the possibility exists that these animals were harbouring a serovar not yet described in New Zealand and not detectable with the panel used in the MAT (DNA sequencing would be needed for confirmation), mice and rats shedding *L*. *borgpetersenii* sv Ballum while having no associated detectable antibodies were already described in New Zealand and remain a more likely explanation. The proportion of ‘silent shedders’ was 67% to 89% in those previous studies (starting dilution 12) [19, 20, 52]. The lower proportion in our sample might be due to a difference in the age ratios. Indeed, when stratified by age in our study, this proportion was 15% (3/20) of adult ‘silent shedders’ and 73% (8/11) of juvenile and subadults. Livestock could similarly be ‘silent shedders’ of Ballum. The presence of overall low titres against this serovar in livestock reinforces this possibility. A nationwide study on dairy farms reported out of 4000 urine samples of adult cows, thirteen (0.3%, 95% CI = 0.2, 0.6) were PCR positive with sequences identified as Ballum, and all were seronegative for Ballum (cut-off 48) [60]. However, it was not possible to investigate the association between shedding and MAT titres in livestock species as part of this study as a result of budgetary constraints and unsuccessful (contaminated) bacterial cultures.

Serovars other than Hardjobovis and Pomona have been largely neglected in surveys conducted on cattle and sheep in NZ since 1983 [18]. Only two recent studies included other “atypical” serovars in their MAT panel and, as we did in this study, they found evidence of exposure in livestock. A nationwide study investigated sera from 1043 beef cattle and 1642 sheep sampled between 2009 and 2010. Seroprevalence for Ballum and Tarassovi in beef cattle was 13.7%, 95% CI [11.7, 16.0%] and 18.0% [15.7, 20.5%] while in sheep it was 10.5% [9.0, 12.1%] and 14.0% [12.4, 15.8%] respectively[61]. In 4000 dairy cattle sampled throughout NZ in 2015, the seroprevalence was 3% [3, 4%] for Ballum and 17% [15, 20%] for Tarassovi [24]. MAT testing in both studies used the same cut-off and was performed in the same laboratory as the current study. In previous surveys conducted between 1967 and 1983, seroprevalence estimates for Ballum in adult cattle were 2.9% (15/520, cut-off 24) [62], 1.3% (8/636, cut-off 20) [63], 3.5% (17/480, cut-off 17) [12], and did not exceed 0.9% at a 100 or 200 cut-off (listed in [18]). Titres were reported for 5/208 calves, representing 2.4% (cut-off 200) [64] but it is unclear whether serology was performed only on those five leptospiruric calves or on all calves sampled.

Cattle from Farm A had been sampled previously after three human cases were reported within three months [28]. That longitudinal study underlined the likely efficiency of vaccination against Hardjobovis and Pomona and antibiotic treatments to reduce shedding in milking cows, previously unvaccinated. It also detected changing dynamics in seroprevalence for other serovars in the replacement stock. In the R1 group there was a low seroprevalence (0 to 2%) to all serovars before vaccination that increased to 73% for Ballum following vaccination, and in the R2 group before vaccination—group not resampled after vaccination—there was a high seroprevalence for Tarassovi only (55% *vs*. 0 to 5% for other serovars) that was not observed in the other groups [28]. In our study, all R1 and R2 had been vaccinated following recommendations [65] and the herd of milking cows was composed of animals first vaccinated at eight months old or more. It is unknown if the lower seroprevalence for Ballum and Tarassovi observed by us in autumn 2017 compared to the seroprevalence measured by Yupiana et al. [28] in summer 2015–2016 are due to vaccination, a seasonal change in exposure to different serovars, a cohort effect linked to annual changes in weather, or another unidentified cause.

The true prevalence of mice exposed to leptospires calculated by occupancy modelling was higher than the apparent prevalence estimated by PCR, culture, or especially MAT, indicating a possible underestimation of the real portion of the population exposed to leptospires. The assumption of a perfect specificity for all tests could artificially increase the difference between observed and true prevalence but true prevalence estimates obtained with Bayesian latent class modelling were similar (S2 Appendix) despite different baseline assumptions for tests specificity (only the PCR specificity set to 100%). The sensitivity of culture and PCR methods partially depends on the bacterial load. When high quantities of *Leptospira* are present in the kidneys, PCR and culture are more likely to give a positive result. The real-time PCR method we used was not quantitative. Although the cycle threshold gives an indication of the concentration of bacterial DNA in the sample, the sampling conducted in remote settings prevented the accurate weighing of the quantity of kidney used for extraction, and therefore the comparison between individuals. Had it been available, an estimate of the bacterial loads would have been helpful in refining the true prevalence occupancy model.

Similar to prevalence, abundance indices of mice and black rats estimated in our study (Table 3) were higher in both Farms A & B than those calculated by Brockie in farm and refuse dump environments in 1974–75 [52]. Expressed in captures per 100 trap-nights (C/100TN), Brockie had respectively 2.97 and 0.76 C/100TN for mice and black rats on farms, and 1.83 and 0.08 C/100TN on refuse dumps [52]. The absence of brown rats in our study suggests they were either absent or present at very low densities in the studied areas. As opposed to black rats, brown rat prevalence was reported to be density-dependent [66]. The density, prevalence and seroprevalence of mice were higher in our study than found by Brockie [52]. This was not observed for black rats, for which numbers were insufficient to rule out a difference. Population dynamics of rodents are known to be cyclical, with rapid changes in densities. To our knowledge, the relationship between *Leptospira* prevalence and species density has had little research attention. In Spain the prevalence of *Leptospira* in micromammals was not related to their relative abundance [67].

Our study demonstrated that livestock were to some extent exposed to serovars that circulate in wildlife in the same environment, especially Ballum in mice. Everywhere those species cohabit, spillover could happen. Farm surroundings often provide ideal habitats for mice, and careful land management would be needed for effective pest control. To understand whether the density of infected rodents likely reflects the risk of spillover to other species, longitudinal studies would be useful.

## Supporting information

S1 Dataset Details of MAT, PCR and culture results used in this study. (XLSX)

S1 Appendix Parameterisation of Occupancy models in E-Surge. (DOCX)

S2 Appendix True prevalence estimation of *Leptospira* infection in mice using Bayesian Latent Class Modelling. (DOCX)

S3 Appendix STROBE Checklist. (DOCX)
