# Supplementary material for: A cross-sectional investigation of Leptospira at the wildlife-livestock interface in New Zealand
Source: PLoS Negl Trop Dis. 2023 Sep 6;17(9):e0011624. doi: 10.1371/journal.pntd.0011624 (PMC10506710; doi:10.1371/journal.pntd.0011624)
Supplement: S1 Appendix — (DOCX) [file pntd.0011624.s002.docx]

## S1 - Occupancy model in E-Surge

Mice were considered as ‘sites’ and laboratory tests as ‘detection occasions’

Assumptions

- All positive tests were regarded as true (no false positive)
- Detection histories were independent
- No change of status during testing

States:

Exposed to *Leptospira* ($\psi)$

Unexposed to *Leptospira* ($1-\psi)$

Dummy-state (‘dead’, needed in GEPAT)

Events:

Tested positive$(p)$

Tested negative$(1-p)$

We defined for model $(\psi,p)$ the initial state $(\Pi)$, transition ($\Phi)$ and event ($B$**)** matrices:

$\Pi=\left[ \begin{matrix} 1-\psi& \psi\end{matrix} \right]$

$\Phi=\left[ \begin{matrix} 1 & 0 \\ 0 & 1 \end{matrix} \right]$ (a specific case of static (single-season) model with no change of state)

$B=\left[ \begin{matrix} 1 & 0 \\ 1-p & p \end{matrix} \right]$

Syntax in GEPAT (GEnerator of PAttern matrices)

$Initial state=\left[ \begin{matrix} * & \pi\end{matrix} \right]$

$Transitions=\left[ \begin{matrix} * & - & - \\ - & * & - \\ - & - & * \end{matrix} \right]$ (the last row and columns are for the ‘dead’ state)

$Event=\left[ \begin{matrix} * & - \\ * & \beta\\ * & - \end{matrix} \right]$ (the last row and columns are for the ‘dead’ state)

Syntax in GEMACO (GEnerator of MAtrices COnstraints)

| Model | (ψ, p) | (ψ_Farm_, p) | (ψ, p_Method_) | (ψ_Farm_, p_Method_) |
| --- | --- | --- | --- | --- |
| Initial state | i | i + g(1,2) | i | i + g(1,2) |
| Transition | i | i | i | i |
| Event | i | i | t | t |
|  |  |  |  |  |

Default parameters in the IVFV menu (Initial Values or Fixed Values of parameters) were used, and the option to compute Hessian confidence intervals selected.
