# Supplementary material for: A cross-sectional investigation of Leptospira at the wildlife-livestock interface in New Zealand
Source: PLoS Negl Trop Dis. 2023 Sep 6;17(9):e0011624. doi: 10.1371/journal.pntd.0011624 (PMC10506710; doi:10.1371/journal.pntd.0011624)
Supplement: S2 Appendix — (DOCX) [file pntd.0011624.s003.docx]

## S2 - True prevalence estimation with Bayesian Latent Class Modelling

In addition to occupancy modelling, true prevalence for each farm was computed using a Bayesian latent class modelling (LCM) for two independent tests and two populations.

The parameters used in the LCM are presented in S2 Table 1. Briefly, this method uses prior knowledge of the parameters and the observed data combined in a likelihood function to simulate a posterior distribution of these parameters via an iterative Markov Chain Monte-Carlo (MCMC) technique. Priors were determined using the literature when available, otherwise one of the co-authors gave their opinion on their value prior to the modelling (S2 Table 1). The MAT and PCR sensitivity and specificity evaluated by Hea [1] were used as priors except the PCR specificity that was assumed to be 100% [2]. Sensitivity and specificity were assumed to be the same in both farms. Prevalence in mice assessed by Hathaway [3] was used as a prior for Farm A, and the prior for prevalence on Farm B suspected to be lower due to the topography of the farm was set to half the value on Farm A. We determined the beta distributions of the priors using the function epi.betabuster in package epiR version 0.9-97 [4]. MAT and PCR measure different outcomes (*Leptospira* DNA in kidney *vs.* antibodies against *Leptospira* in blood), and titres are a poor predictor of shedding status [5]. We made therefore no adjustment for correlation. We ran 10,000 MCMC iterations and discarded the first 5000. We extracted from the posterior distributions the means and 2.5^th^ and 97.5^th^ percentiles to build the 95% credible intervals. To assess convergence, we used the last 5000 iterations in chain to plot a Gelman-Rubin-Brooks graph [6]. A sensitivity analysis was conducted using weak and perturbed priors (weakly informative (Jeffrey’s prior), high (80%) or low (1%) priors for prevalence and optimistic (99%) or pessimistic (55%) priors for test parameters, S2 Table 1) for each parameter and magnitude and direction of changes in the posterior distributions were noted. LCM were run in ℝ with package R2OpenBUGS version 3.2-3.2 [7] and OpenBUGS version 3.2.3. Package coda version 0.19-2 [8] was used for convergence diagnosis. Details on the R code used and diagnostics conducted are presented below.

S2 Table 1 | Parameters used in the Latent Class Model

| A |  |  |  | B |  |  |  |  |
| --- | --- | --- | --- | --- | --- | --- | --- | --- |
| Farm A | PCR – | PCR + |  |  | "best guess" | 95% certain | α | β |
| MAT – | 48 | 8 |  | π_A_ | 0.16^a^ | < 0.5^c^ | 2.04 | 6.47 |
| MAT + | 3 | 15 |  | π_B_ | 0.08^c^ | < 0.5^c^ | 1.36 | 5.12 |
| Farm B | PCR – | PCR + |  | Se_PCR_ | 0.65^b^ | > 0.6^c^ | 171.39 | 92.75 |
| MAT – | 27 | 1 |  | Se_MAT_ | 0.84^b^ | > 0.5^c^ | 6.47 | 2.04 |
| MAT + | 0 | 5 |  | Sp_MAT_ | 0.73^b^ | > 0.7^c^ | 466.48 | 173.16 |
|  |  |  |  | Jeffrey's | \ | \ | 0.50 | 0.50 |
|  |  |  |  | High π | 0.8 | < 0.9 | 17.62 | 5.16 |
|  |  |  |  | Low π | 0.01 | < 0.1 | 1.34 | 34.17 |
|  |  |  |  | Opt Se/Sp | 0.99 | > 0.95 | 88.28 | 1.88 |
|  |  |  |  | Pes Se/Sp | 0.55 | > 0.3 | 5.99 | 5.08 |
| A- Contingency tables for the number of mice tested by Microscopic Agglutination Test (MAT) and lipL32 PCR on Farm A and B.  B-Determination of the beta prior distribution (α, β) for the 5 parameters to be estimated: the true prevalence on Farm A and B (πA, πB), the sensitivity of each diagnostic test (SePCR, SeMAT) and the MAT specificity (SpMAT), and perturbed values used in the sensitivity analysis: weakly informative (Jeffrey’s prior), high (80%) or low (1%) priors for prevalence and optimistic (99%) or pessimistic (55%) priors for test parameters. PCR sensitivity was assumed to be 1. Estimations based on ^a^ [3], ^b^ [1], ^c^expert opinion | | | | | | | | |

The estimate of true prevalence in mice given by the LCM was 36.8% [95% Credible Interval 25.2-49.6%] on Farm A and 21.8% [9.8-37.6%] on Farm B. Although the current study did not aim at estimating the sensitivity and specificity of the tests used, the model estimates of MAT sensitivity and specificity were 62.6% [45.9-78.1%] and 75.4% [72.1-78.5%], and the estimate of PCR sensitivity was 66.9% [61.4-72.2%]. Results of the LCM sensitivity analysis can be visualized in the forest plot (S2 Figure 1). Applying weakly informative priors for prevalence on Farm A or B did not substantially affect the posterior median values of prevalence (+2%), and extreme change (increase to 80% or decrease to 1%) in the priors only lead to a moderate change (same direction) in the posterior values (+ 16% and – 12% for Farm A, + 29% and – 10% for Farm B).


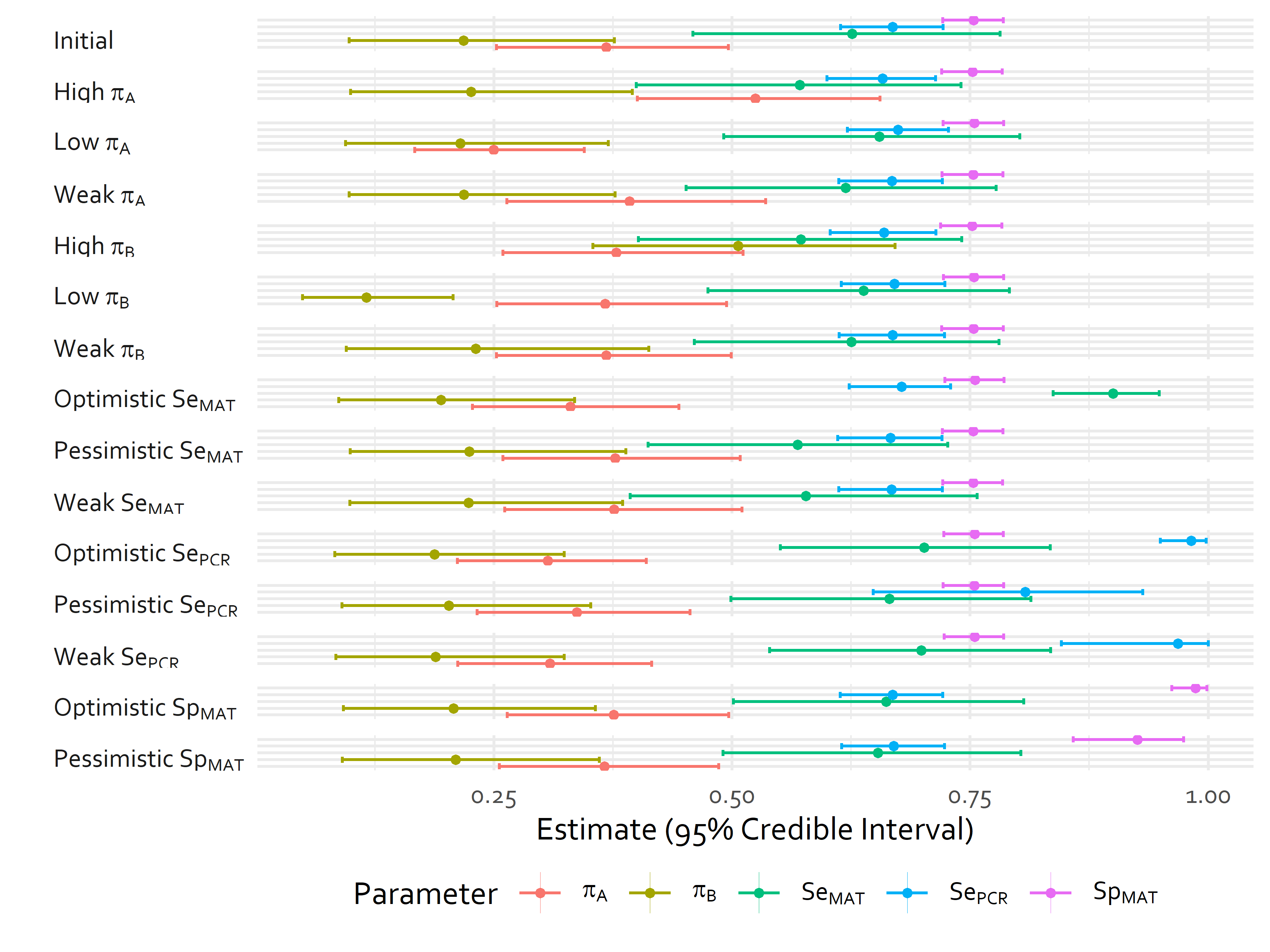


S2 Figure 1 | Forest plot showing the parameters and their credible interval estimated by the initial latent class model and the other models run as a sensitivity analysis.

The five estimated parameters are the true prevalence on Farm A and B (π_A_, π_B_), the sensitivity of each diagnostic test (Se_PCR_, Se_MAT_) and the MAT specificity (Sp_MAT_)Weakly informative (Jeffrey’s prior), high (80%) or low (1%) priors for prevalence and optimistic (99%) or pessimistic (55%) priors for test parameters were used in the sensitivity analysis.

R Code and diagnostics run

The code in the Diagnostics section was inspired from Chi Yau, Bayesian Inference Using OpenBUGS [ cited 28 Nov 2022]. In R Tutorials [Internet]. Available from <http://www.r-tutor.com/bayesian-statistics/openbugs>

#load libraries
library(readxl)

library(epiR) # for epi.betabuster()

library(R2OpenBUGS) # for bugs()

library(coda) # for bugs(codaPkg=TRUE) and to read coda files/make graphs

library(lattice)# for graphs with coda files
library(dplyr) # for bind_rows and other

library(stringr) # for strg_replace
library(tibble) # for add_column

library(ggplot2) # for forest plot

###determination of priors Beta distribution (BetaBuster)
Priors <- read_excel("Priors.xlsx")
Pmode<-Priors$mode
Pconf<-Priors$conf
Pgreaterthan<-Priors$greaterthan
Px<-Priors$x

a<-c()
b<-c()

for ( i in seq_along(Priors)) {

 tmp<-epi.betabuster(mode = Pmode[i], conf = Pconf[i], greaterthan = Pgreaterthan[i], x = Px[i], conf.level = 0.95, max.shape1 = 1000, step = 0.001)
 a<-c(a,tmp$shape1)
 b<-c(b,tmp$shape2)
}
result <- data.frame(a,b)
write.table(result,"clipboard",sep="\t", row.names = FALSE)
#and ctrl+V in excel spreadsheet

## LCM

#initial model
model <- function() {
 # Priors
 #Dairy
 piA ~ dbeta(2.04, 6.47)

 #Beef
 piB ~ dbeta(1.36, 5.12)

 #PCR
 SePCR ~ dbeta(171.39, 92.75)
 #SpPCR ~ (0.97+0.03)

 #MAT
 SeMAT ~ dbeta(6.47, 2.04)
 SpMAT ~ dbeta(466.48, 173.16)

 # Likelihood
 #Dairy
 X1[1:4] ~ dmulti(p1[1:4], n1)
 p1[1]<-piA*SePCR*SeMAT #11
 p1[2]<-piA*SePCR*(1-SeMAT) #10
 p1[3]<-piA*(1-SePCR)*SeMAT +(1-piA)*(1-SpMAT) #01
 p1[4]<-piA*(1-SePCR)*(1-SeMAT) + (1-piA)*SpMAT #00

 #Beef
 X2[1:4] ~ dmulti(p2[1:4], n2)
 p2[1]<-piB*SePCR*SeMAT #11
 p2[2]<-piB*SePCR*(1-SeMAT) #10
 p2[3]<-piB*(1-SePCR)*SeMAT +(1-piB)*(1-SpMAT) #01
 p2[4]<-piB*(1-SePCR)*(1-SeMAT) + (1-piB)*SpMAT #00

}
model.file <- file.path(tempdir(), "model.txt")
write.model(model, model.file)

data <- list(X1=c(15, 8, 3, 48), X2=c(5, 1, 0, 27), n1=74, n2=33)
params <- c("piA","piB","SePCR", "SeMAT","SpMAT")
inits <- function() { list(piA=0.16,piB=0.08,SePCR=0.65, SeMAT=0.84,SpMAT=0.73) }

out <- bugs(data, inits, params, model.file, n.iter=10000)
all(out$summary[,"Rhat"] < 1.1) #if false, increase n.iter

## [1] TRUE

print(out, digits=5)

## Inference for Bugs model at "model.txt",
## Current: 3 chains, each with 10000 iterations (first 5000 discarded)
## Cumulative: n.sims = 15000 iterations saved

## mean sd 2.5% 25% 50% 75% 97.5%
## piA 0.36761 0.06227 0.25239 0.32420 0.36500 0.40862 0.49591
## piB 0.21803 0.07213 0.09766 0.16570 0.21220 0.26370 0.37620
## SePCR 0.66862 0.02770 0.61380 0.65010 0.66910 0.68740 0.72150
## SeMAT 0.62596 0.08240 0.45880 0.57127 0.62825 0.68300 0.78140
## SpMAT 0.75376 0.01632 0.72110 0.74280 0.75400 0.76510 0.78500
## deviance 59.29163 3.75102 52.61000 56.68000 59.06000 61.55000 67.47050
## Rhat n.eff
## piA 1.00104 14000
## piB 1.00121 6500
## SePCR 1.00092 15000
## SeMAT 1.00130 5000
## SpMAT 1.00116 7700
## deviance 1.00128 5200
##
## For each parameter, n.eff is a crude measure of effective sample size,
## and Rhat is the potential scale reduction factor (at convergence, Rhat=1).
##
## DIC info (using the rule, pD = Dbar-Dhat)
## pD = 2.56200 and DIC = 61.85000
## DIC is an estimate of expected predictive error (lower deviance is better).

##Diagnostics
out <- bugs(data, inits, params, model.file, codaPkg=TRUE, n.iter=10000)
out.coda <- read.bugs(out)

xyplot(out.coda) #check if simulation values stabilize


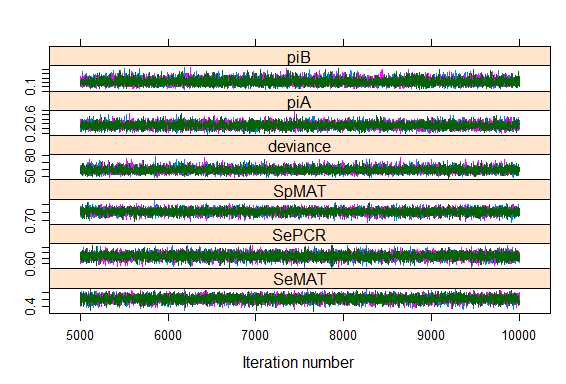


densityplot(out.coda) #inspect if the density plot is well-defined


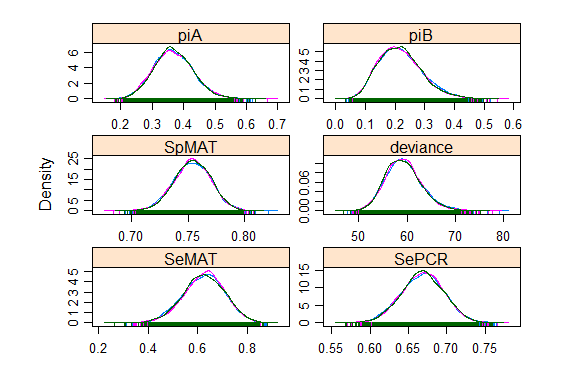


acfplot(out.coda) #check if the auto-correlation of the time series converge to zero


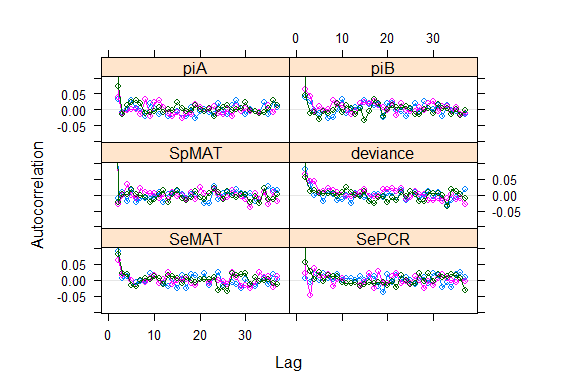

gelman.diag(out.coda) #shrink factors should be below 1.05

## Potential scale reduction factors:
##
## Point est. Upper C.I.
## SeMAT 1 1
## SePCR 1 1
## SpMAT 1 1
## deviance 1 1
## piA 1 1
## piB 1 1
##
## Multivariate psrf
##
## 1

gelman.plot(out.coda) #Gelman-Rubin-Brooks plot for visual confirmation of the shrink factor convergence


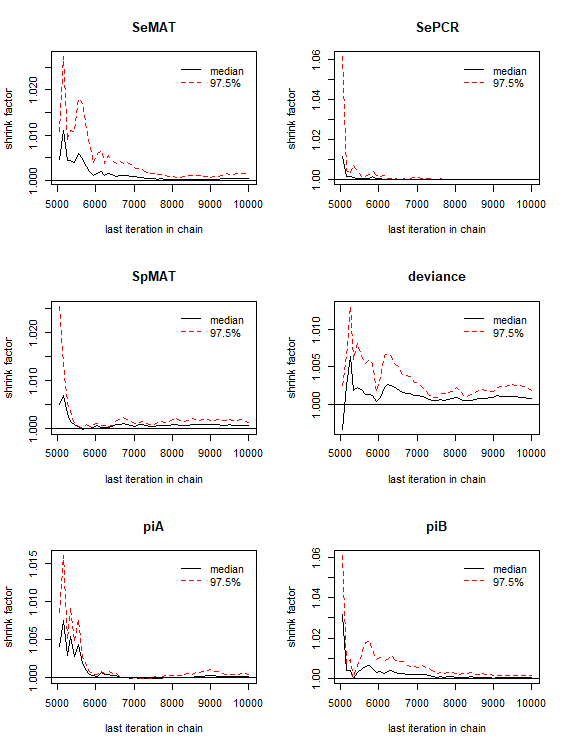


#retrieve point estimates and 95% credible intervals
out.summary <- summary(out.coda, q=c(0.025, 0.975))
out.summary$stat

## Mean SD Naive SE Time-series SE
## SeMAT 0.6259558 0.08240187 0.0006728085 0.0007279397
## SePCR 0.6686218 0.02770335 0.0002261969 0.0002336840
## SpMAT 0.7537568 0.01631514 0.0001332126 0.0001323422
## deviance 59.2916307 3.75101593 0.0306269168 0.0339350688
## piA 0.3676077 0.06227121 0.0005084423 0.0005417730
## piB 0.2180274 0.07212884 0.0005889295 0.0006331803

out.summary$q

## 2.5% 97.5%
## SeMAT 0.45879750 0.7814025
## SePCR 0.61379750 0.7215025
## SpMAT 0.72110000 0.7850000
## deviance 52.61000000 67.4705000
## piA 0.25239500 0.4959075
## piB 0.09765975 0.3762000

Run<-cbind(out.summary$stat,out.summary$q)
write.csv(Run,file = "Run_initial.csv", row.names = TRUE)

References

1. Hea S-Y. A Bayesian approach to the estimation of the sensitivity and specificity of diagnostic tests for leptospirosis, and the estimation of infection prevalence in sheep and cattle. [M.V.S. thesis]. Palmerston North, NZ: Massey University; 2014.

2. Stoddard RA, Gee JE, Wilkins PP, McCaustland K, Hoffmaster AR. Detection of pathogenic *Leptospira* spp. through TaqMan polymerase chain reaction targeting the *LipL32* gene. Diagn Micr Infec Dis. 2009;64(3):247-55. doi: https://doi.org/10.1016/j.diagmicrobio.2009.03.014. PubMed PMID: WOS:000267277300002.

3. Hathaway SC, Blackmore DK, Marshall RB. Leptospirosis in free-living species in New Zealand. Journal of wildlife diseases. 1981;17(4):489-96. Epub 1981/10/01. PubMed PMID: 7338970.

4. Stevenson M, Nunes T, Heuer C, Marshall JC, Sanchez J, Thornton R, et al. epiR: Tools for the Analysis of Epidemiological Data. 2018.

5. Fang F, Collins-Emerson JM, Heuer C, Hill FI, Tisdall DJ, Wilson PR, et al. Interlaboratory and between-specimen comparisons of diagnostic tests for leptospirosis in sheep and cattle. Journal of Veterinary Diagnostic Investigation. 2014;26(6):734-47. Epub 2014/10/09. doi: https://doi.org/10.1177/1040638714548476. PubMed PMID: 25292194.

6. Brooks SP, Gelman A. General methods for monitoring convergence of iterative simulations. Journal of Computational and Graphical Statistics. 1998;7(4):434-55.

7. Sturtz S, Ligges U, Gelman A. R2WinBUGS: A Package for Running WinBUGS from R. Journal of Statistical Software. 2005;12(3):1-16.

8. Plummer M, Best N, Cowles K, Vines K. CODA: Convergence Diagnosis and Output Analysis for MCMC. R News. 2006;6(1):7-11.
