# Supplementary material for: A cross-sectional investigation of Leptospira at the wildlife-livestock interface in New Zealand
Source: PLoS Negl Trop Dis. 2023 Sep 6;17(9):e0011624. doi: 10.1371/journal.pntd.0011624 (PMC10506710; doi:10.1371/journal.pntd.0011624)
Supplement: S3 Appendix — (DOCX) [file pntd.0011624.s004.docx]

STROBE Statement—checklist of items that should be included in reports of observational studies

|  | Item No. | Recommendation | Page  No. | Relevant text from manuscript |
| --- | --- | --- | --- | --- |
| **Title and abstract** | 1 | (*a*) Indicate the study’s design with a commonly used term in the title or the abstract | 1 | Cross-sectional in the title |
|  |  | (*b*) Provide in the abstract an informative and balanced summary of what was done and what was found | P1-2 | Trapped wild animals and an age-stratified random sample of domestic animals, namely cattle, sheep and working dogs were blood sampled. Sera were tested by microagglutination test for five serogroups and titres compared using a Proportional Similarity Index (PSI). Wildlife kidneys were sampled for culture and qPCR targeting the *lipL32* gene. True prevalence in mice was assessed using occupancy modelling by collating different laboratory results.  Infection profiles varied by species, age group and farm. At the MAT cut-point of ≥ 48, up to 78% of wildlife species, and 16 to 99% of domestic animals were seropositive. Five hedgehogs (n=9), 23 mice (n=105) and one black rat (n=14) reacted to *L. borgpetersenii* sv Ballum. Four of both the possums (n=18) and the hedgehogs (n=9) reacted to *L. borgpetersenii* sv Hardjobovis whilst another possum and another hedgehog reacted to Tarassovi. In ruminants, seroprevalence for Hardjobovis and Pomona ranged 0-90% and 0-71% depending on the species and age group. Titres against Ballum, Tarassovi and Copenhageni were also observed in 4-20%, 0-25% and 0-21% of domestic species respectively. The PSI indicated rodents and livestock had the most dissimilar serological responses. Three of nine hedgehogs, 31/105 mice and 2/14 rats were carrying leptospires (PCR and/or culture positive). True prevalence estimated by occupancy modelling in mice was respectively 38% [95% Credible Interval 26, 51%] on Farm A and 22% [11, 40%] on Farm B. |
| Introduction | | | |  |
| Background/rationale | 2 | Explain the scientific background and rationale for the investigation being reported | P3-4 | 5 first paragraphs of the introduction |
| Objectives | 3 | State specific objectives, including any prespecified hypotheses | P4 | “It is unknown if this change in livestock and human epidemiology is due to an increased exposure to maintenance hosts shedding those emerging serovars or a change in the role of livestock in maintaining them. Up-to-date information on all serovars circulating in wildlife and livestock is needed.  The objectives of this cross-sectional study were to (1) estimate the seroprevalence in wildlife and sympatric livestock in two farm environments; (2) estimate PCR/culture prevalence in wildlife on those farms; (3) compare seroprevalence and prevalence between species, sex and age groups in the two farms, and (4) estimate the true prevalence of natural *Leptospira* infection in mice using occupancy models.” |
| Methods | | | |  |
| Study design | 4 | Present key elements of study design early in the paper | P4-6 | Mention of a cross-sectional study at the end of the introduction  Description of the populations sampled and mention of the point in time at which the cross-section was taken |
| Setting | 5 | Describe the setting, locations, and relevant dates, including periods of recruitment, exposure, follow-up, and data collection | P4-5  P5-7 | Paragraph on Study sites  For wildlife: “traps were set in March-April 2017 for up to 10 days per site”  For livestock: Sample dates in Table 1 |
| Participants | 6 | (*a*) *Cohort study*—Give the eligibility criteria, and the sources and methods of selection of participants. Describe methods of follow-up  *Case-control study*—Give the eligibility criteria, and the sources and methods of case ascertainment and control selection. Give the rationale for the choice of cases and controls  *Cross-sectional study*—Give the eligibility criteria, and the sources and methods of selection of participants | P6 | “Trapped wild animals”  “We sampled healthy domestic animals by mob according to the farmers’ schedule (when animals were gathered for milking, drenching, shearing, pregnancy testing or annual vaccine booster). We expected a seroprevalence of 20% for dogs (which was the seroprevalence observed in working dog breeds [35]), of 80% in sheep and beef cattle (which was the seroprevalence observed in beef & sheep farms when *Leptospira* was present [36], and 50% in dairy cattle (which was the seroprevalence in the dairy farm at first sampling [26]). Assuming the proportions of seropositive animals in each farm and group were as expected, and adjusting for a finite population, we used the formula for estimating the expected seroprevalence with 10% absolute precision and 95% confidence in [37] to calculate the sample size in each species or age-group (Table 1).” |
|  |  | (*b*) *Cohort study*—For matched studies, give matching criteria and number of exposed and unexposed  *Case-control study*—For matched studies, give matching criteria and the number of controls per case |  | Not applicable |
| Variables | 7 | Clearly define all outcomes, exposures, predictors, potential confounders, and effect modifiers. Give diagnostic criteria, if applicable | P9 | “We differentiated the seroprevalence (estimated by MAT), the prevalence (estimated by culture or PCR) and the true prevalence (the proportion of animals exposed to *Leptospira* infection). The probability of shedding amongst seropositive and seronegative wild animals was calculated by dividing the number of animals positive for PCR and/or culture by the total number of animals tested within each stratum.” |
| Data sources/ measurement | 8* | For each variable of interest, give sources of data and details of methods of assessment (measurement). Describe comparability of assessment methods if there is more than one group | *P8-9* | *“All samples were processed by the same laboratory personnel.“* |
| Bias | 9 | Describe any efforts to address potential sources of bias | P9  P10 | - - - - 1. “**True prevalence**. Misclassification bias can arise from the use of imperfect tests giving false-positive and false-negative results. To limit this bias, true prevalence in mice was computed for each farm using site-occupancy modelling”         2. **“Kappa test for cross-reaction.** Agreement beyond chance between MAT results for different serovars was tested using Kappa (κ) tests for all wild and all domestic species.” |
| Study size | 10 | Explain how the study size was arrived at | P11  P6-7 | For wildlife  “There were respectively 720, 430 and 351 trap-nights for Longworth, Tomahawk and Havahart traps on Farm A. Because of an accident while setting the traps on Farm B, those figures were slightly lower on Farm B (648, 418, 332). Rats, hedgehogs and possums were trapped in both Tomahawk and Havahart traps. The number of animals captured per 100 trap-nights and sampled in each farm are detailed in Table 3.”  For livestock  We expected a seroprevalence of 20% for dogs (which was the seroprevalence observed in working dog breeds [35]), of 80% in sheep and beef cattle (which was the seroprevalence observed in beef & sheep farms when *Leptospira* was present [36], and 50% in dairy cattle (which was the seroprevalence in the dairy farm at first sampling [26]). Assuming the proportions of seropositive animals in each farm and group were as expected, and adjusting for a finite population, we used the formula for estimating the expected seroprevalence with 10% absolute precision and 95% confidence in [37] to calculate the sample size in each species or age-group (Table 1). |

Continued on next page

| Quantitative variables | 11 | Explain how quantitative variables were handled in the analyses. If applicable, describe which groupings were chosen and why | P8  P9 | The endpoint of an agglutination reaction was deemed to be the dilution at which approximately 50% of Leptospira had agglutinated and expressed as a reciprocal titre (e.g. titre 24 for dilution 1:24). Since this serological test was used to assess previous exposure (seroprevalence) to leptospires at the population level, and not for clinical diagnosis, the positive threshold was set at a titre of 48 or higher [39].   - - - - 1. “**Geometric Mean Titres (GMT)**   . The geometric mean titre of positive sera (GMT) and all sera (GMT0) was calculated using the formulas given in [36]. While sera for which no antibodies were detected (titre <24) were excluded from the calculation of the GMT, they were given a log-titre of 0 and included in the calculation of the GMT0.” |
| --- | --- | --- | --- | --- |
| Statistical methods | 12 | (*a*) Describe all statistical methods, including those used to control for confounding | P9-11 | description of methods used for  Confidence Intervals for proportions  True prevalence  Kappa test for cross-reaction  PSI-Czekanowski index |
|  |  | (*b*) Describe any methods used to examine subgroups and interactions | P9-10 | Cf True prevalence estimated for mice (only species with sufficient numbers) |
|  |  | (*c*) Explain how missing data were addressed | P10 | Mice with missing data for at least one laboratory method were not included. |
|  |  | (*d*) *Cohort study*—If applicable, explain how loss to follow-up was addressed  *Case-control study*—If applicable, explain how matching of cases and controls was addressed  *Cross-sectional study*—If applicable, describe analytical methods taking account of sampling strategy |  | Not applicable |
|  |  | (*e*) Describe any sensitivity analyses |  | In supplementary materials S2 |
| Results | | | | |
| Participants | 13* | (a) Report numbers of individuals at each stage of study—eg numbers potentially eligible, examined for eligibility, confirmed eligible, included in the study, completing follow-up, and analysed | P7 P12  P13 | In Table 1, Table 3 and Table 4 |
|  |  | (b) Give reasons for non-participation at each stage | P11 | “We did not have the opportunity to sample the R1 and mixed-age beef cattle on Farm B during the study” |
|  |  | (c) Consider use of a flow diagram |  |  |
| Descriptive data | 14* | (a) Give characteristics of study participants (eg demographic, clinical, social) and information on exposures and potential confounders |  | Results stratified by species, farm and (for livestock) age |
|  |  | (b) Indicate number of participants with missing data for each variable of interest | P12 | In Table 3 |
|  |  | (c) *Cohort study*—Summarise follow-up time (eg, average and total amount) |  | Not applicable |
| Outcome data | 15* | *Cohort study*—Report numbers of outcome events or summary measures over time |  | *Not applicable* |
|  |  | *Case-control study—*Report numbers in each exposure category, or summary measures of exposure |  | *Not applicable* |
|  |  | *Cross-sectional study—*Report numbers of outcome events or summary measures |  | *In Table 3 and 4* |
| Main results | 16 | (*a*) Give unadjusted estimates and, if applicable, confounder-adjusted estimates and their precision (eg, 95% confidence interval). Make clear which confounders were adjusted for and why they were included | P12  P16 | Unadjusted estimates in Table 3 and Table 4 with 95% CI (for groups >5 animals)  Estimates of true prevalence adjusted for unperfect detection for mice in the text |
|  |  | (*b*) Report category boundaries when continuous variables were categorized |  | Not applicable |
|  |  | (*c*) If relevant, consider translating estimates of relative risk into absolute risk for a meaningful time period |  | Not applicable |

Continued on next page

| Other analyses | 17 | Report other analyses done—eg analyses of subgroups and interactions, and sensitivity analyses | P10 | “True prevalence was also estimated using a second method, Bayesian latent class modelling and the results compared (S2 Appendix).” |
| --- | --- | --- | --- | --- |
| Discussion | | | | |
| Key results | 18 | Summarise key results with reference to study objectives | P17 | “Prior to this current study, the most recent studies at the wildlife-livestock interface in NZ were conducted in the late 1980s, four decades ago. These concluded that “despite high prevalence of endemic infection of Hardjobovis and Pomona in cattle and pigs respectively and Ballum and Balcanica in wildlife, [there was] virtually no evidence of interspecies transmission” [22, p. 111]. Today, whether due to changes in vaccination or farming practices, in ecological factors, species distributions or diagnostic techniques, we can see that this is no longer the case. Antibodies against serovars detected in wildlife in our study were commonly found in livestock sharing the same environment” |
| Limitations | 19 | Discuss limitations of the study, taking into account sources of potential bias or imprecision. Discuss both direction and magnitude of any potential bias | P19 | The interpretation of titres in dogs was further hindered by the absence of information on the vaccine type used for working dogs. Although all vaccines licensed for dogs in NZ only cover the serogroup Icterohaemorrhagiae (*i.e.* sv Copenhageni), it is possible farmworkers administered an off-label cattle trivalent vaccine (Hardjo, Pomona and Copenhageni) on dogs from Farm B as this practice is suspected to be common for working dogs [52, 53]. Titres against Hardjo in possums were likely indicative of an exposure to Balcanica, another serovar in serogroup Hebdomadis that this species harbours [18], but there were no isolates to confirm the infecting serovar.  In a cross-sectional study like this, the timing of infection is unknown, and animals previously infected with antibodies titres below the detection limit cannot be distinguished from animals never exposed [56].  However, due to budgetary constraints and contamination of urine samples from livestock, it was not possible in the current study to investigate the association between shedding and MAT titres in livestock species. |
| Interpretation | 20 | Give a cautious overall interpretation of results considering objectives, limitations, multiplicity of analyses, results from similar studies, and other relevant evidence | P17 | “Antibodies against serovars detected in wildlife in our study were commonly found in livestock sharing the same environment, supporting the concept of inter-species disease transmission, or spillover” |
| Generalisability | 21 | Discuss the generalisability (external validity) of the study results | P17  P21 | “The small number of serovars known to circulate in NZ allows a better interpretation of *Leptospira* inter-host species epidemiology than in other countries where *Leptospira* diversity is higher.“  “Everywhere those species cohabit, spillover could happen.” |
| Other information | |  | | |
| Funding | 22 | Give the source of funding and the role of the funders for the present study and, if applicable, for the original study on which the present article is based |  | In the Financial Disclosure Statement |

*Give information separately for cases and controls in case-control studies and, if applicable, for exposed and unexposed groups in cohort and cross-sectional studies.

**Note:** An Explanation and Elaboration article discusses each checklist item and gives methodological background and published examples of transparent reporting. The STROBE checklist is best used in conjunction with this article (freely available on the Web sites of PLoS Medicine at http://www.plosmedicine.org/, Annals of Internal Medicine at http://www.annals.org/, and Epidemiology at http://www.epidem.com/). Information on the STROBE Initiative is available at www.strobe-statement.org.
